# Exposure of domestic dogs and cats to ticks (Acari: Ixodida) and selected tick-borne diseases in urban and recreational areas in southern Poland

**DOI:** 10.1038/s41598-022-11973-4

**Published:** 2022-05-12

**Authors:** Anna Kocoń, Marek Asman, Magdalena Nowak-Chmura, Joanna Witecka, Grzegorz Rączka

**Affiliations:** 1 Department of Zoology, Institute of Biology, Pedagogical University of Cracow, Podchorążych 2, 30-084 Cracow, Poland; 2 Department of Parasitology, Faculty of Pharmaceutical Sciences in Sosnowiec, Medical University of Silesia, Jedności 8, 41-218 Sosnowiec, Poland; 3 Department of Forest Management Planning, Poznań University of Life Sciences, Wojska Polskiego 71c, 60‑625 Poznań, Poland

**Keywords:** Entomology, Microbial ecology, Parasitology, Pathogens

## Abstract

The public health problem of tick-borne diseases has attracted much attention in recent years due to an increasing incidence in humans and animals. The aim of this study was to compare the risk of exposure to ticks and tick-borne infections in dogs and cats in recreational and urbanized areas in the Lesser Poland and Silesian Provinces. For molecular testing for the presence of the selected pathogens, 207 *I*. *ricinus* females collected from 119 dogs and 50 cats, and 2 *I*. *hexagonus* females collected from 2 domestic dogs, were examined. Overall, *A*. *phagocytophilum* was found in 3.7% of the *I*. *ricinus* specimens, *B*. *microti* in 27.1%, and *B*. *burgdorferi* s.l. in 0.9%. In urban areas of both provinces, *A*. *phagocytophilum* was found in 4.8% of the *I*. *ricinus* specimens, *B*. *microti* in 41.6% *and* *B*. *burgdorferi* s.l. in 3.9%*.* Pathogens were detected *B*. *microti* in both studied *I*. *hexagonus* specimens. These findings may indicate the important role that these animals play in the circulation of these pathogens in nature.

## Introduction

Ticks, as one of the most common external parasites in domestic cats and dogs, play an important role in the natural transmission of pathogens between hosts. The high adaptability of ticks to changing environmental and weather conditions, as well as the large host range and the ability to transmit pathogens transstadially and transovarially, contribute to the spread of these parasites and pathogens over ever larger areas and to companion animals. The high density of ticks in forest biotopes, which constitute a natural source of pathogen infections, is conditioned by the high diversity of mammalian species. In addition, due to the migration of hosts on which ticks feed, increased tourism and the more frequent travel of owners with companion animals, ticks are transferred from their natural forest habitat to other areas that provide them with access to a larger host range. In this way, new disease entities can appear in areas where they previously did not occur^1^.

Frequent travel by people with their pets is a likely contributor to the existing epizootic situation for many external parasites and the pathogens they transmit. According to a 2017 survey in Poland, more than half of Poles (52%) have a pet in their household. Residents of rural areas and small towns are more likely to own pets. The most common animal in Polish homes is the domestic dog (*Canis* *familiaris*; 42%), followed by the domestic cat (*Felis* *catus*; 26%), while both a dog and a cat were owned by 18% of those surveyed^2^.

The large number of human cases of tick-borne diseases that have been reported in Central Europe confirms the need for research regarding the relationships between hosts, especially between humans and their pets. Dogs and cats may play a significant role in the transmission cycles of many agents of vector-borne diseases by serving as amplifying hosts^3,4^. Many of these pet vector-borne pathogens can also affect humans due to their zoonotic potential. In view of the great medical and veterinary significance of ticks and the close contact between humans and pets, an attempt was made to compare the occurrence and determine the level of exposure of dogs and cats to tick infestations, as well as to determine the prevalence of selected pathogens in these parasites, including *Borrelia* *burgdorferi* s. l., *Anaplasma* *phagocytophilum*, *Babesia* *microti* and *Toxoplasma* *gondii*, in selected recreational and urbanized habitats in the Lesser Poland and Silesian Provinces.

## Materials and methods

The area of southern Poland (the Lesser Poland and Silesian Provinces) is rich in places for rest and recreation, such as hiking trails, forests, and river valleys, and has abundant fauna and flora, which encourage residents and tourists to spend their free time on walks, excursions, and family picnics with their pets, and, consequently, to be exposed to ticks.

The Lesser Poland Province is situated in southern Poland and spans across parts of the Western Carpathians and the Lesser Poland Upland, bordering with Slovakia. The topography is mountainous and upland in character. In terms of landscape, it is a very diverse area. In the south, there are the Tatra Mountains, the Beskids and the limestone Pieniny, from west to east the Vistula River flows, and the Krakow-Czestochowa Upland is situated in the north. The hydrographic network consists almost exclusively of rivers belonging to the upper Vistula and Czarna Orawa drainage basins. The plant and animal world is rich, which is evidenced by the presence of as many as 6 National Parks and 11 Landscape Parks in the area. Beech and spruce forests, as well as fragments of primeval forest, are preserved in the Pieniny Mountains, Babia Góra and the Tatra Mountains, with forests occupying 28.6% of the total area of the Lesser Poland region. Owing to its natural, cultural and historical heritage, Lesser Poland has a high tourism potential^5^.

The Silesian Province, on the other hand, bordering the Lesser Poland Province from the west, is characterized by great geographical and landscape diversity. Here too can be found mountainous, upland and lowland areas. These include the Żywiec and Silesian Beskids, the Beskidian Foothills, the wooded areas of the Silesian Lowlands and the urbanized area of the Silesian Uplands. In the eastern part of the province is the Kraków–Częstochowa Upland. Silesia borders on the Czech Republic and Slovakia. The watershed that runs through the province separates the basins of the Vistula and Odra, and, in the south, the Vistula and Danube. Within the urbanized areas, in contrast to the surrounding areas, the formation of local topoclimates associated with anthropogenic factors affecting temperature and precipitation can be observed. In spite of its industrial character, Silesia also possesses many valuable environmental and landscape attributes, which contribute to increasing tourism. Forests cover about 32% of the area, and 8 Landscape Parks have been established in the area. The forests found here are mostly comprised of pine, spruce and oak^6^. Both provinces are among the largest industrial, economic, tourist, spa, academic, historical and cultural centers, and, as a result, tourists often visit these areas, spending their leisure time actively or passively in the open air, thereby facilitating the spread of parasites, including ticks, to other areas.

**Figure 1:**
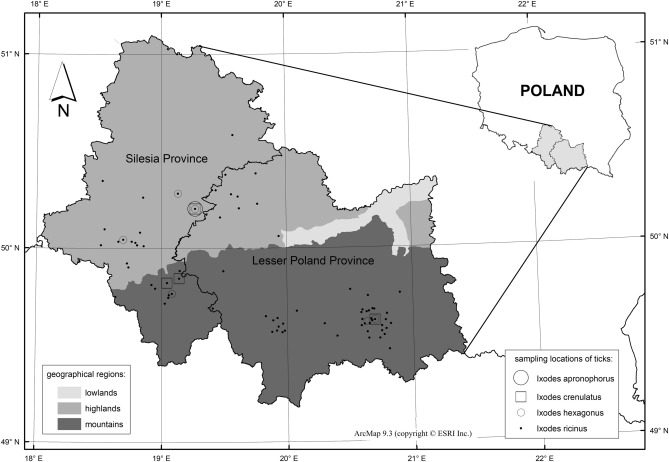
The locations of collected tick species in the selected areas in the Lesser Poland and Silesian Provinces. The maps were created using licensed software ArcMap ver. 9.3 ( copyright ESRI Inc.).

Ticks were collected from domestic dogs and cats between February and October 2017 in cooperation with veterinary clinics. These clinics were located in urbanized (Olkusz, Żory, Jaworzno, and Sosnowiec) and tourist cities (Nowy Sącz, Wadowice, Rabka Zdrój, Bielsko-Biała and Cieszyn) in the Lesser Poland and Silesian Provinces. Specimens were also collected from an animal shelter in Sosnowiec (Fig. 1). The ticks were collected from the animals with tweezers and placed into test tubes with 70% ethanol. A proprietary form was filled out after collection, which included the date of collection, the breed of the animal, its sex and age, and the city. The ticks were then labelled with the genus, species, and the developmental stage according to the criteria of Siuda^7^ and Nowak-Chmura^8^.

For molecular tests, ticks were selected from characteristic recreational and urbanized sites/cities, with diverse terrain and different access to hosts were selected in order to compare the presence of tick-borne disease pathogens. DNA was isolated from individual ticks using the ammonia method^9^, and its concentration was measured spectrophotometrically at 260/280 nm. Pathogens in the tested material were detected by PCR and nested PCR. For the detection of *B.* *burgdorferi* s.l., a pair of primers specific for the flagellin gene and Maximo DFS-Plus polymerase (GeneOn, Germany) were used^10^. Two pairs of primers specific for the 16S rRNA gene and Taq DNA polymerase (EURx, Poland) were used to detect *A.* *phagocytophilum*^11^. The protozoa *B.* *microti* and *T.* *gondii* were detected using two pairs of primers specific for the 18S rRNA gene using Maximo DFS-Plus polymerase (GeneOn, Germany) and the B1 gene using Taq DNA polymerase (EURx, Poland), respectively^12,13^. Amplification and re-amplification products were separated electrophoretically in 2% agarose gels and stained with ethidium bromide. The gels were then visualized under ultraviolet light. The presence of reaction products with 482 base pairs [bp] for *B.* *burgdorferi* s. l., 932 bp and 546 bp for *A.* *phagocytophilum*, 238 bp and 154 bp for *B.* *microti* and 531 bp for *T.* *gondii* were treated as positive samples.

To verify the significance of differences in the levels of infestation, the average number of ticks parasitizing dogs and cats in the different groups were compared using Mann–Whitney U tests. In turn, verification of the significant differences in the degree of tick infestation was performed using the *Z*-test for the two independent proportions in several grouping factors: species of domestic mammals, administrative provinces, geographical regions and climatic seasons. To test the significance of differences in the number of ticks parasitizing dogs and cats, and the number of ticks infected and not infected with the pathogens in the different groups, the χ^2^ test was used. All statistical analyses were performed using STATISTICA software and the alpha level was set at p < 0.05 for all tests.

### Ethical approval and consent to participate

We declare that all testing methods have been carried out in accordance with the relevant guidelines and regulations. We declare that all experimental protocols have been approved by the Medical University of Silesia in Katowice and Pedagogical University in Cracow.

Since the study was carried out provided voluntarily by dog and cat owners (including the director and veterinary services of a local dog and cat shelter), no ethical approval/license was required for our study (as per Resolution on the protection of animals used for scientific or educational purposes, 15th January 2015 [Dz. U. 2015 position 266] Chapter 1, Paragraph 1.2.1). The owners of dogs involved in this study were informed about the aims of the study, provided oral consent and contact information to obtain the results of testing.

## Results

A total of 909 adult ticks and 2 nymphs, representing four species, were collected from 469 domestic animals. The species observed were *Ixodes* *apronophorus* (*n* = 1), *Ixodes* *crenulatus* (*n* = 12), *Ixodes* *hexagonus* (*n* = 36) and *Ixodes* *ricinus* (*n* = 864). The range of the most abundant species (*I.* *ricinus*) covered the entire study area, while the occurrence of the other tick species were limited to single locations (Fig. 1).

**Figure 2:**
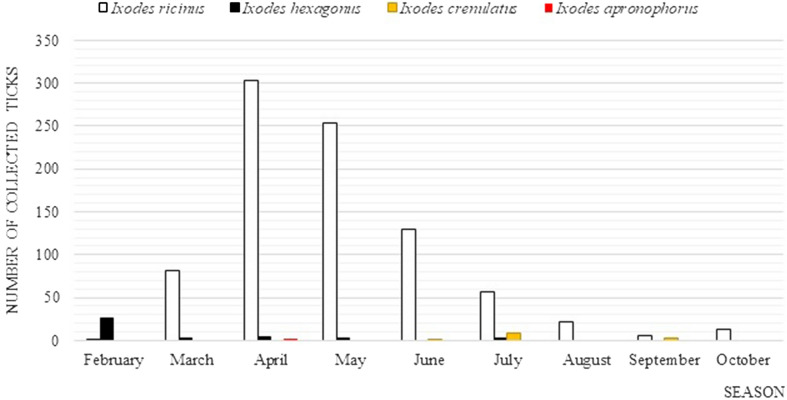
Activity of collected ticks from pets in the Lesser Poland and Silesian Provinces.

In the case of *I.* *ricinus*, infestations on domestic animals were most frequent in April and May, and least frequent in February and September. The tick *I.* *hexagonus* was found in the highest numbers in February, while its presence was the least frequent in March, May and July. *I*. *crenulatus* was most frequently found on the animals in July and least frequently in June, and the female *I*. *apronophorus* was only found in April (Fig. 2).

Overall, the average number of ticks parasitizing a single cat (*avg* = 2.415) was significantly higher than that of a single dog (*avg* = 1.942; *U* = 1.781, *p* = 0*.*05).

**Figure 3:**
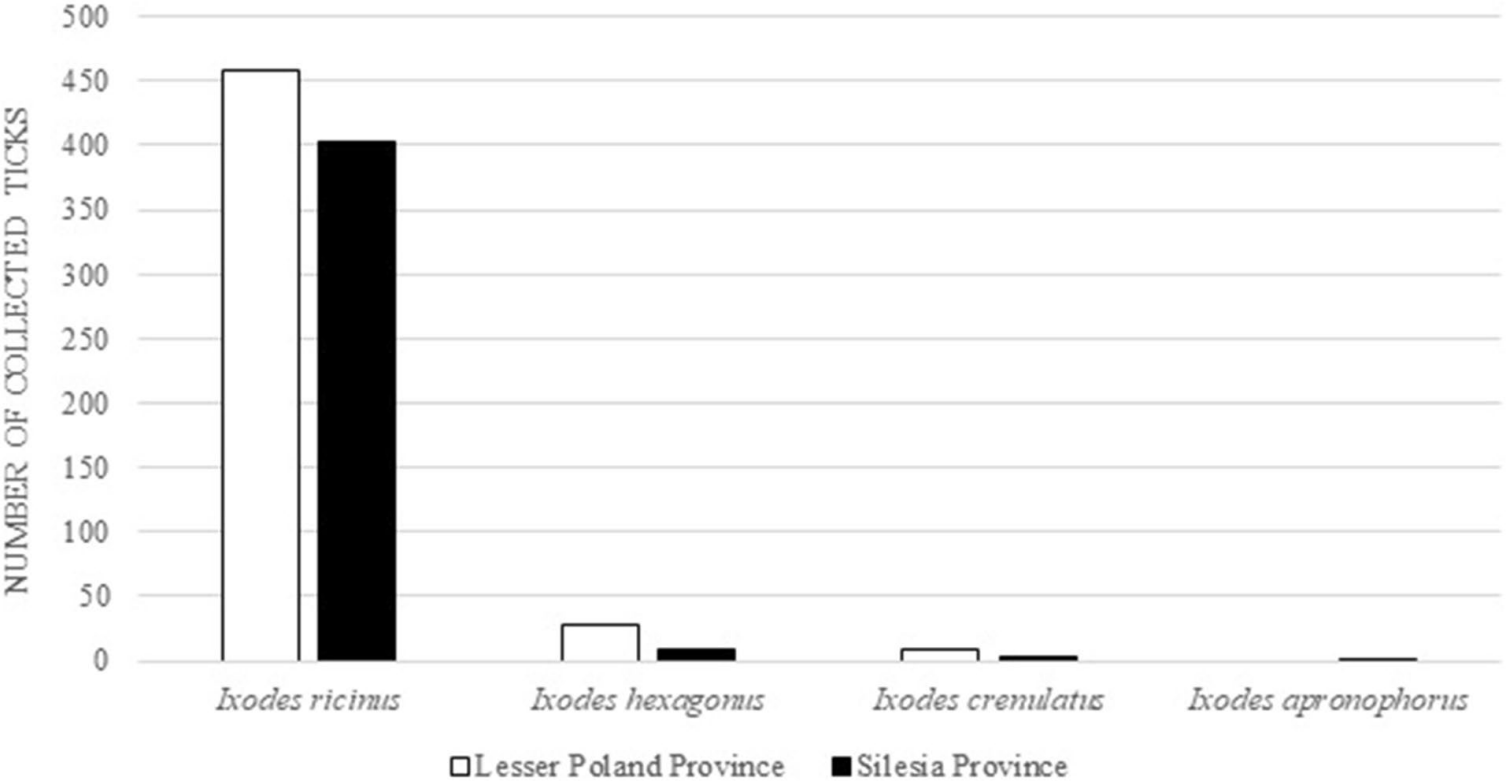
The total number of tick specimens collected from pets in the study areas in the Lesser Poland and Silesian Provinces.

Across the region of Lesser Poland, a total of 491 ticks were collected from 168 pet dogs and 47 pet cats. The ticks collected in this region included *I*. *ricinus* (n = 459, 407 females, 52 males), *I*. *hexagonus* (n = 27, 26 females and 1 male), and *I*. *crenulatus* (n = 9 females). In the Silesian Province, a total of 420 ticks were collected from 171 dogs and 83 cats, including *I*. *ricinus* (n = 402, 371 females, 20 males and 1 nymph), *I*. *hexagonus* (n = 9, 7 females, 1 male and 1 nymph), *I*. *crenulatus* (n = 3 females), and *I*. *apronophorus* (n = 1 female; Fig. 3).

Spatially, a significantly higher average number of ticks was found on cats (*avg* = 2.590) than on dogs (*avg* = 1.247) in Silesia (*U* = 3.062, *p* = 0.005) and in the uplands (*U* = 3.101, *p* = 0.005), where the average number of ticks feeding on cats was 2.761 and on dogs was 1.205. In contrast, in the Lesser Poland Province, the average number of ticks parasitizing a single cat (*avg* = 2.063) was found to be lower than that of a single dog (*avg* = 2.190). This was similar to the mountainous regions where, on average, more ticks were found on dogs (*avg* = 2.329) than on cats (*avg* = 1.967), but these differences were not statistically significant. The χ^2^ tests showed significant differences between the number of ticks parasitizing cats and dogs, depending on the analyzed area (i.e., province or geographical region). The number of ticks feeding on cats was lower in the Lesser Poland Province than in the Silesian Province, and the number of ticks feeding on dogs was higher in the Lesser Poland province than in the Silesia Province, compared to expected numbers (*χ*^*2*^ [1, *N* = 909] = 108.9, *p*-value < 0.001). Similar significant differences were also found for the geographical regions, with ticks infesting cats being less abundant in mountainous than in upland areas, and ticks feeding on dogs being more abundant in mountainous than in upland areas, compared to the expected numbers (*χ*^*2*^ [1, *N* = 909] = 64.4, *p* < 0.001).

**Figure 4:**
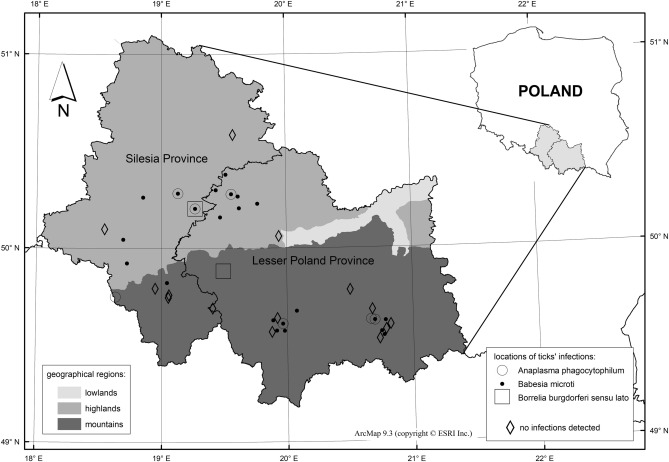
Locations of the occurrence of tick-borne disease pathogens in selected research areas in the Lesser Poland and Silesian Provinces. The maps were created using licensed software ArcMap ver. 9.3 ( copyright ESRI Inc.).

A total of 209 female ticks, collected at 39 sites, were included in the molecular studies, with 14 sites showing no presence of the tested pathogens and the remaining 25 sites showing the presence of at least one of the pathogens. *Anaplasma* *phagocytophilum* was found in ticks in 7 locations, *B.* *microti* in 22, and *B.* *burgdorferi* s.l. only in 2 (Fig. 4). In case no *T*. *gondii* was detected in the examined ticks.

In total, pathogens were found in 44.0% of the examined ticks, including in 43.5% of the *I.* *ricinus* specimens and in 2 female of the *I*. *hexagonus* specimens (Table 1). Out of the 100 female *I*. *ricinus* ticks collected from 72 dogs and 20 cats in the Lesser Poland Province, the most common microorganism was *B*. *microti* (47.0%), followed by *A*. *phagocytophilum* (4.0%), and *B*. *burgdorferi* s. l. (2.0%). There were also 2 co-infections with *A*. *phagocytophilum* and *B*. *microti* (2.0%), and 1 with *B*. *microti* and *B*. *burgdorferi* s. l. (1.0%). In the Silesian Province, out of the 107 female *I*. *ricinus* specimens from 47 dogs and 30 cats that were examined, the most common microorganism was *B*. *microti* (22.5%), followed by *A*. *phagocytophilum* (4.7%) and *B*. *burgdorferi* s. l. (4.7%). *B*. *microti* was also detected in the 2 female *I*. *hexagonus* specimens collected from 2 dogs (100.0%; Table 1).

**Table 1:** The total numbers and percentages of female *Ixodes* *ricinus* and *Ixodes* *hexagonus* ticks infected with *Anaplasma* *phagocytophilum*, *Babesia* *microti* or *Borrelia* *burgdorferi* in the study areas in the Lesser Poland and Silesian Voivodeships.

Collecting site	Tick species	Number of studied ticks	1 pathogen			2 pathogens	
			Ap	Bm	Bbsl	Ap + Bm	Bm + Bbsl
Lesser Poland Province	*Ixodes* *ricinus*	100	4 (4.0%)	47 (47.0%)	2 (2.0%)	2 (2.0%)	1 (1.0%)
Silesia Province	*Ixodes* *ricinus*	107	5 (4.7%)	24 (22.5%)	5 (4.7%)	0 (0.0%)	0 (0.0%)
	*Ixodes* *hexagonus*	2	0 (0.0%)	2 (100.0%)	0 (0.0%)	0 (0.0%)	0 (0.0%)
Total		209	9 (4.3%)	73 (35.0%)	7 (3.3%)	2 (1.0%)	1 (0.5%)

*Ap *Anaplasma* *phagocytophilum*, *Bm* *Babesia* *microti*, *Bbsl* *Borrelia* *burgdorferi* sensu lato.

Overall, in the Lesser Poland Province, selected tick-borne disease pathogens were found in 56.0% of the *I*. *ricinus* females. The highest number of infected ticks from this area was found in domestic dogs and cats from the veterinary clinics in Olkusz (77.0% *B*. *microti*, 3.3% *B*. *burgdorferi* s.l.) and Rabka Zdrój (53.3% *B*. *microti*, 3.3% *A*. *phagocytophilum*). Single cases of *B*. *microti* (28.6%) and *A*. *phagoctophilum* (7.1%) were reported in Nowy Sącz, and one case of *B*. *burgdorferi* s.l. (8.3%) in Wadowice. Two co-infections were also found in Olkusz (*A*. *phagoctophilum* and *B*. *microti* [3.3%], and *B*. *microti* and *B*. *burgdorferi* s.l. [3.3%]) and one in Nowy Sącz (*A*. *phagoctophilum* and *B*. *microti* [3.6%]; Table 2).

**Table 2:** The total numbers and percentages of *Ixodes* *ricinus* females infected with *Anaplasma* *phagocytophilum*, *Babesia* *microti* and *Borrelia* *burgdorferi* in the surveyed areas in the Lesser Poland Voivodeship.

Collecting site	Number of studied ticks	Monoinfection			Co-infection	
		Ap	Bm	Bbsl	Ap + Bm	Bm + Bbsl
1	30	1 (3.3%)	23 (77.0%)	1 (3.3%)	1 (3.3%)	1 (3.3%)
2	12	0 (0.0%)	0 0 (0.0%)	1 (8.3%)	0 (0.0%)	0 0 (0.0%)
3	28	2 (7.1%)	8 (28.6%)	0 0 (0.0%)	1 (3.6%)	0 0 (0.0%)
4	30	1 (3.3%)	16 (53.3%)	0 0 (0.0%)	0 0 (0.0%)	0 0 (0.0%)
Total	100	4 (4.0%)	47 (47.0%)	2 (2.0%)	2 (2.0%)	1 (1.0%)

*1—Olkusz; 2—Wadowice; 3—Nowy Sącz; 4—Rabka Zdrój; *Ap* *Anaplasma* *phagocytophilum*, *Bm* *Babesia* *microti*, *Bbsl* *Borrelia* *burgdorferi* sensu lato.

In contrast, in the examined ticks from the Silesian Province, a total of 31.0% of females were infected with tick-borne pathogens (*I*. *ricinus* 30% and *I*. *hexagonus* 100%). Among the domestic dogs and cats from veterinary clinics, the highest numbers of *I*. *ricinus* females infected with *B*. *microti* were found in Żory city (75.0%), Bielsko-Biała (29.4%), and Jaworzno (14.5%). Among the 2 *I*. *hexagonus* specimens, one case each of *B*. *microti* was found from the animal shelter in Sosnowiec (100.0%) and in Żory (100.0%). Single cases of *A*. *phagocytophilum* were found in Sosnowiec (16.7%), Jaworzno (6.2%) and Bielsko-Biała (5.0%), while *B*. *burgdorferi* s. l. was detected in *I*. *ricinus* females in Jaworzno (6.2%; Table 3).

**Table 3:** The total numbers and percentages of *Ixodes* *ricinus* and *Ixodes* *hexagonus* ticks infected with *Anaplasma* *phagocytophilum*, *Babesia* *microti* and *Borrelia* *burgdorferi* in the surveyed areas in the Silesian Voivodeship.

Collecting site	Tick species	Number of studied ticks	Pathogen		
			Ap	Bm	Bbsl
1	*Ixodes* *ricinus*	6	1 (16.7%)	0 (0.0%)	0 (0.0%)
	*Ixodes* *hexagonus*	1	0 (0.0%)	1 (100.0%)	0 (0.0%)
2	*Ixodes* *ricinus*	48	3 (6.2%)	7 (14.5%)	3 (6.2%)
3	*Ixodes* *ricinus*	16	0 (0.0%)	12 (75.0%)	0 (0.0%)
	*Ixodes* *hexagonus*	1	0 (0.0%)	1 (100.0%)	0 (0.0%)
4	*Ixodes* *ricinus*	17	0 (0.0%)	5 (29.4%)	0 (0.0%)
5	*Ixodes* *ricinus*	20	1 (5.0%)	0 (0.0%)	0 (0.0%)
Total		110	5 (4.5%)	26 (23.7%)	3 (2.8%)

*1—Sosnowiec; 2—Jaworzno; 3—Żory; 4—Bielsko-Biała; 5—Cieszyn. *Ap* *Anaplasma* *phagocytophilum*, *Bm* *Babesia* *microti*, *Bbsl* *Borrelia* *burgdorferi* sensu lato.

The results of the significance tests carried out for two independent proportions of tick infections by pathogens indicate highly significant differences in the values of these proportions in two cases for *B.* *microti*: by province and in relation to the seasons when ticks parasitizing pets were collected. The proportion of *B.* *microti* infections in the Lesser Poland Province (z = 0.500) was significantly higher than in the Silesian Province (z = 0.239; p < 0.001), and in the spring period (z = 0.918) it was significantly higher than in the summer-autumn period (*z* = 0.135; *p* < 0.001). In the remaining analyzed cases, which also included other pathogens (*B.* *burgdorferi* s.l., and *A.* *phagocytophilum*), in relation to the host species (cat, dog) and geographical regions (highlands, mountains), the differences were insignificant (Table 4).

**Table 4:** Values of the test statistics for two structure indicators (*z*) of tick infections by pathogens (*Anaplasma* *phagocytophilum*, *Babesia* *microti*, *Borrelia* *burgdorferi*) in different comparison groups (species of domestic mammal, administrative province, geographical region, and climatic season).

Grouping factor	Pathogen		
	Ap	Bm	Bbsl
Species of domestic mammals: Cat Dog	0.223	1.789	1.087
Administrative provinces: Silesia Province Lesser Poland Province	0.209	3.925*	0.356
Geographical regions: Highlands Mountains	0.385	1.883	1.391
Climatic seasons: Spring Summer/autumn	0.280	10.696*	1.453

*The result is significant at *p* < .001, *Ap* *Anaplasma* *phagocytophilum*, *Bm* *Babesia* *microti*, *Bbsl* *Borrelia* *burgdorferi* sensu lato.

The results of the χ^2^ test, which was carried out to compare the number of ticks infected and not infected with *B.* *microti* protozoa, turned out to be significant only in relation to the province. The number of ticks vectoring this protozoan was higher in the Lesser Poland Province than in the Silesian Province, while the number of ticks not vectoring *B.* *microti* was higher in the Silesian Province than in the Lesser Poland Province, compared to the expected numbers (*χ*^2^ [1, *N* = 209] = 15.4, *p* < 0.001). In contrast, the numbers of infected and uninfected *B.* *microti* ticks did not differ significantly in the other comparison groups (host species, geographical region and climatic season).

## Discussion

Of the 19 species of ticks that are permanently present in Poland, domestic dogs are attacked by four species of Ixodidae and one species of Amblyommidae, including *Ixodes* *ricinus*, *Ixodes* *crenulatus*, *Ixodes* *hexagonus*, *Ixodes* *rugicollis* (Schulze and Schlottke, 1929), and *Dermacentor* *reticulatus*^8,14,15^ and own unpublished research. Cases of *Rhipicephalus* *sanguineus* (Latreille, 1806) infesting domestic dogs have been reported^16^; however, this species is not a component of the natural Polish tick fauna. Nonetheless, a mass occurrence of this species in a Warsaw flat has been reported^16^. In contrast, domestic cats are infested by four species of the family Ixodidae, including *Ixodes* *ricinus*, *Ixodes* *crenulatus*, *Ixodes* *hexagonus*, and *Ixodes* *rugicollis*^17–19^. Domestic dogs and cats may additionally be affected by tick species such as *Ixodes* *persulcatus* (Schulze, 1930), *Haemaphysalis* *punctata* (Canestrini and Fanzago, 1878) and *Haemaphysalis* *concinna* (Koch, 1844)^8,14^.

To date, few studies have been conducted in the Lesser Poland and Silesian Provinces to investigate the risk of pet exposure to ticks and the importance of these hosts as reservoirs of tick-borne pathogens^15,18,20–23^. Several studies conducted in Europe and Poland indicate that the dominant tick species infecting domestic dogs and cats is *I.* *ricinus*^17,24–31^. The current faunistic analysis carried out in two provinces in southern Poland also showed a dominance of this tick species with regard to infestations in these animals.

Numerous cases of canine Lyme disease have been reported throughout Europe^32^, with many cats not showing signs of the disease^33^. Lyme disease in dogs is a condition of great concern in veterinary practice due to the fact that it is zoonotic. Canine Lyme disease has now been found in north-western Poland and in the Lublin region^34–36^. It is estimated that only about 5% of infested dogs are symptomatic^37^. The presence of Lyme disease in cats has not yet been established in Poland. The presence of *B.* *burgdorferi* s.l. in *I*. *ricinus* collected from domestic animals in Poland varies from single digits to up to 10.5% in south-eastern Poland^19,38,39^. A significantly higher value was obtained by researchers in Olsztyn, who demonstrated the presence of this bacterium in 31.6% of *I*. *ricinus* specimens collected from dogs^40^. In contrast, studies in Europe indicate a slightly higher proportion of ticks infected with *B*. *burgdorferi* s. l. In Germany, Schreiber et al.^27^ found this spirochete in 11.6% of ticks, while studies in the Netherlands and Belgium found *B.* *burgdorferi* s. l. in 7.2% and 10.1% of *I.* *ricinus* specimens, respectively^26,41^. Single cases of *B*. *burgdorferi* s. l. infections have also been observed in domestic animals in Portugal^3^. The results obtained in the current study are lower than those obtained from western Europe and south-eastern Poland. There were also no significant differences in the number of ticks infected with this bacterium between the two studied provinces. The low occurence of this bacterium in ticks from upland and highland areas indicates that there is a relatively low risk of exposure of dogs and domestic cats to the presence of this bacterium in ticks in these areas regardless of the region.

Canine babesiosis, although well recognized by veterinarians, presents many problems with diagnosis and treatment. To date, only infections with the subspecies *Babesia* *canis* *canis* have been detected in Poland (Welc-Falęciak, 2009). Adaszek et al. (2011) have shown that the Lublin, Podlasie and Masovian Provinces are most at risk from canine babesiosis; however, an increasing number of cases of this disease are being reported in the Western Provinces^39,42^. In cats, babesiosis is mainly caused by the protozoan *Babesia* *felis*. First case of this disease in cats described by Adaszek et al.^43^. Król et al.^17^ identified the presence of this protozoan in 9.0% of ticks collected from domestic dogs and cats. In contrast, a study conducted in selected mountainous areas by Kocoń et al.^44^ showed a more than twofold higher percentage of ticks infected with this protozoan that were collected from pets. The results obtained in the current study show a relatively high rate of infection with *B.* *microti* (35.0%) among the ticks examined. Significance tests for two independent proportions of tick infections by pathogens indicate highly significant differences in the values of these proportions in two cases (administrative provinces and climatic seasons) for *B.* *microti*. A similar value was obtained by researchers working in the Tarnogórski district (42.6%)^45^. Results from other European countries have demonstrated the presence of *B.* *microti* in 1.4–8.0% of ticks collected from domestic dogs^27,46,47^. *Babesia* *microti* occurs sporadically; therefore, this study may have revealed a higher proportion of infected ticks. The differences in prevalence suggest possible local effects involving vector distribution or density, which may condition the exposure of dogs and cats to tick-borne diseases.

Feline granulocytic anaplasmosis is relatively rare in Europe, but cases have been reported in Sweden, Finland, Denmark, Ireland, the UK, Italy, Germany, and Switzerland^48–55^. In Poland, infection with this rickettsia has been documented in cats from Lublin, Przemyśl and Mazovia^56–59^. Anaplasmosis in dogs in Europe is currently observed in e.g. Switzerland, the UK, and Sweden^60–62^. In Poland, data on this subject are sparse; however, the presence of the etiological agent for this disease has been reported in eastern Poland^63,64^. The incidence of *A*. *phagocytophilum* in the examined specimens was 4.0% in the Lesser Poland Province and 4.7% in the Silesian Province. No significant differences in the number of infected ticks were observed between the two studied provinces. Similar results were obtained in central and south-eastern Poland, where the percentage of ticks infected with *A.* *phagocytophilum* ranged from 0.96–6%^19,38,40^. The values obtained in the current study are significantly lower than those obtained by Król et al.^17^, which showed the presence of this rickettsia in 21.3% of the examined *I.* *ricinus* ticks collected from dogs and cats in the Wrocław metropolitan area. Similar studies in domestic pets conducted in several European countries reported a higher percentage of *I.* *ricinus* specimens infected with *A.* *phagocytophilum* (17.0%)^30^, and in Belgium 19.5%. In the Czech Republic and the Netherlands, a low percentage of ticks with *A.* *phagocytophilum* (3.4%) has been reported^41,65^. The low prevalence of *A.* *phagocytophilum* in the examined specimens may be due to the generally low proportion of ticks infected with this bacterium in the study area, which may be related to the geographical location or the limited access of ticks to hosts due to the selection of urban areas for the study.

There are also known cases of the co-occurrence of two or three pathogens in *I.* *ricinus* ticks collected from domestic animals^17,45^. In a study conducted in the Tarnogórski district, Asman et al.^45^ demonstrated the co-occurrence of *B.* *microti* and *T.* *gondii* in more than 40% of the examined *I.* *ricinus* ticks collected from dogs and cats. King et al.^17^ also showed the co-occurrence of 2 or even 3 pathogens in a single *I.* *ricinus* tick, with *A.* *phagocytophilum* and *Rickettsia* spp. being the most frequently found in cases of dual infection. In contrast, in ticks collected from dogs in the Tatra district, the co-occurrence of *A.* *phagocytophilum* and *B.* *microti,* and *A.* *phagocytophilum* and *T.* *gondii*, was observed in only two *I.* *ricinus* females^44^. The results of these studies confirm the possibility of co-infection with *A*. *phagocytophilum* and *B*. *microti*, as well as *B*. *burgdorferi* s. l. and *B*. *microti*, in ticks collected from pet dogs. This finding suggests the possibility of co-infections in animal with more than one pathogen, which can make diagnosis difficult in the case of symptoms. The failure to demonstrate pathogen coexistence in ticks collected from cats may be related to the lower number of ticks collected from these animals.

## Conclusions

Surveys indicate that, in the Lesser Poland and Silesian Provinces, domestic dogs and cats are mainly infested by ticks of the species *I.* *ricinus*. However, they can also be attacked by *I.* *hexagonus*, *I.* *crenulatus* and *I.* *apronophorus*, but such occurrences are infrequent. The study area was also shown to have a potentially risk of *B.* *microti*, *A.* *phagocytophilum* and *B.* *burgdorferi* s.l. tick infection. It cannot be excluded, however, that some specimens may have been pathogen-positive due to feeding on an infected (asymptomatic) animal. Overall, the current results indicate that domestic companion animals may contribute to the circulation of ticks and pathogens in both recreational and urban areas. The results also suggest the existence of two likely infestation channels in the study areas. The first is female ticks feeding on cats in the upland part of the Silesian Province. The other is female ticks feeding on dogs in the mountainous part of the Lesser Poland Province. In conclusion, both recreational and urban areas in the study region can be favorable habitats for ticks, which justifies the regular testing of companion animals for ticks and tick-borne pathogens in order to provide information on the potential risks to humans.

## Data Availability

The data presented in this study are contained within the article.
